# Evaluation of a Pilot Digital Antibiotic Tool

**DOI:** 10.1111/tct.70428

**Published:** 2026-04-16

**Authors:** James Bowen, Johnathan Mollman, Daniel Herchline, Bindu Alex, Sarah Pickering, Sonya Tang Girdwood

**Affiliations:** ^1^ Division of Pediatric Hospital Medicine Cincinnati Children's Hospital Medical Center Cincinnati Ohio USA; ^2^ Department of Pediatrics University of Cincinnati College of Medicine Cincinnati Ohio USA; ^3^ Pediatric Residency Program Cincinnati Children's Hospital Medical Center Cincinnati Ohio USA; ^4^ Division of Pharmacy Cincinnati Children's Hospital Medical Center Cincinnati Ohio USA; ^5^ Division of Translational and Clinical Pharmacology Cincinnati Children's Hospital Medical Center Cincinnati Ohio USA

**Keywords:** antibiotics, graduate medical education, just‐in‐time learning

## Abstract

**Background:**

Selecting and prescribing antibiotics are essential skills for paediatric residents, yet many report low confidence and variability in education on this topic. Traditional reference tools are often comprehensive but not optimised for rapid, point‐of‐care consultation or easy customisation. To address these challenges, we developed a digital educational resource tailored to common antibiotic prescribing needs in residency.

**Approach:**

A multidisciplinary team of paediatric hospitalists, pharmacists and residents created a website featuring concise guidance for 13 commonly used antibiotics. Antibiotic selection was informed by a needs assessment survey and institutional data on pharmacist‐led interventions for resident antibiotic orders. The tool emphasised clarity and usability rather than comprehensiveness. Users could access dosing, contraindications and institutional guidance without logging in, on either mobile or desktop platforms.

**Evaluation:**

Over 6 months, the website received 1100 visits, including 734 from self‐identified paediatric residents. The three most frequently visited antibiotic pages aligned with the three antibiotics that most often required pharmacist modification (cefazolin, amoxicillin and amoxicillin‐clavulanate). In a follow‐up survey (response rate: 42%), residents rated the site's usability as above average using the System Usability Scale (mean score, 77.3, *n* = 22). Free‐text feedback highlighted ease of use, relevance and utility for teaching peers.

**Implications:**

This learner‐informed digital tool demonstrated strong early engagement and usability. The alignment between prescribing challenges and site usage suggests feasibility to assist clinical practice. Future directions include integrating the tool into the electronic health record (EHR) and assessing effects on prescribing accuracy, user knowledge, and patient safety across contexts.

## 1 Background

Infections represent a leading cause of paediatric hospitalisation, making antibiotic prescribing practices essential for physicians [1]. In paediatrics, infections such as pneumonia, urinary tract infection and sepsis are some of the most common and most costly diagnoses requiring hospitalisation [2]. This importance of selecting appropriate antimicrobials is highlighted in the Accreditation Council for Graduate Medical Education requirements for paediatric residents [3] and the American Board of Paediatrics general certification examination content specifications [4]. While antibiotic education is deliberate and structured in undergraduate medical education, it becomes less standardised in paediatric residency, where learning is often experiential and variable [5, 6].

Currently, paediatric residents often rely on online databases, textbooks and institutional guidelines for antibiotic prescribing. While comprehensive and evidence‐based, these resources are primarily designed for clinical reference rather than for graduate medical education. Their expansive nature, while valuable for consultation, is not aligned with adult learning principles that emphasise accessibility, efficiency and applicability for self‐directed learning [7, 8]. Clinical work demands require residents to make prescribing decisions quickly, making easy‐to‐use references paramount in supporting these decisions. Residents may benefit from institutional resources that consolidate locally relevant prescribing guidance (based on formulary and local organism susceptibilities) in a concise, rapidly accessible format. Implementation science literature emphasises that successful integration of digital resources depends on content quality in addition to usability, workflow alignment, accessibility and user acceptability [9, 10, 11]. Before evaluating educational impact, it is necessary to determine whether such a resource is feasible to implement and acceptable to users within existing clinical workflows. Therefore, we sought to create a resource to address residents' needs in antibiotic prescribing and assessed its usability and feasibility to be integrated into clinical practice.

*Clinical work demands require residents to make prescribing decisions quickly, making easy‐to‐use references paramount in supporting these decisions.*

## 2 Approach

This IRB‐exempt study was performed at a large, freestanding, academic children's hospital comprised of over 670 beds. There is an infectious disease service for consultation in addition to dedicated inpatient pharmacists who are present on rounds for certain subspecialty teams. There is an antimicrobial stewardship team primarily focused on judicial use of broad‐spectrum antibiotics. Currently, the primary online resource available for antibiotic prescribing is a comprehensive database (Lexicomp). We conducted a needs assessment [12] (Table 1) via a voluntary, anonymous, online survey to explore resident perspectives regarding antibiotic selection to guide construction of an antibiotic prescribing resource. Medical education experts reviewed survey questions, and five cognitive interviews were performed with residents outside of our sample to enhance construct and response process validity. The survey was disseminated to categorical paediatric residents (*n* = 98) via e‐mail and completed by 57% of recipients (*n* = 56). Results illustrated that 54% (*n* = 30) of residents did not feel comfortable in selecting antibiotics, and 96% (*n* = 54) of respondents noted they would use a digital tool to learn about antibiotics.

**TABLE 1:** A brief needs assessment disseminated to categorical paediatric residents prior to website development.

Pre‐implementation needs assessment (*n* = 56)	
Post‐graduate year (PGY)	
1	21 (38%)
2	16 (29%)
3	19 (34%)
State your agreement: I am comfortable in selecting an antibiotic regimen for common paediatric infections.	
Strongly disagree, disagree, neutral	30 (54%)
Agree, strongly agree	26 (46%)
Would you use a digital tool to assist in selecting antibiotic regimens?	
Yes	54 (96%)
No	2 (4%)

**FIGURE 1:**
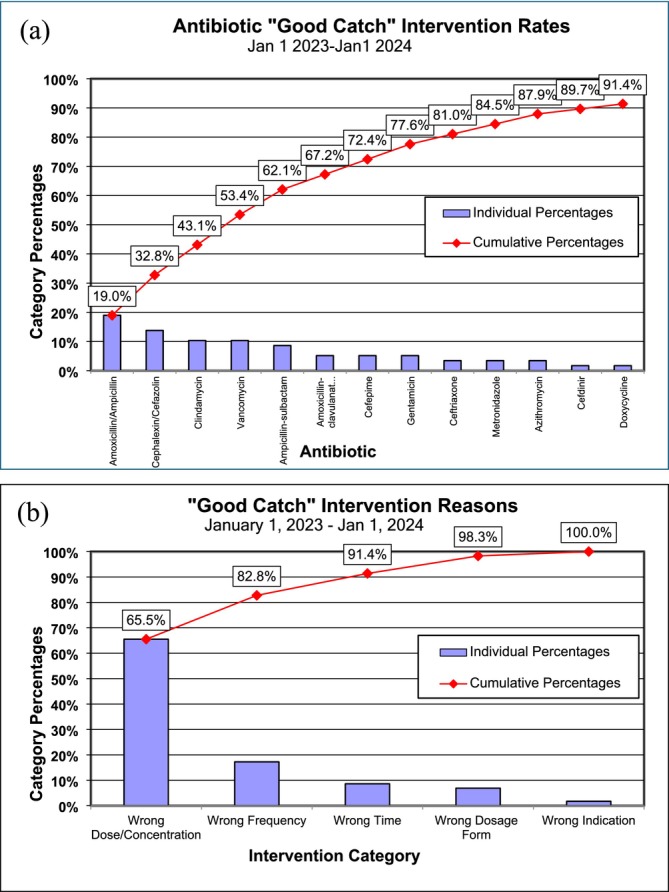
Pareto chart illustrating types of antibiotics (a) intervened upon and (b) reasons for pharmacist intervention.

Our institution maintains a repository documenting pharmacist‐led interventions to orders prior to dispensing of medications and includes medication ordered and reasons for intervention. Despite the availability of Lexicomp, antibiotics were the drug class requiring the most pharmacist‐led interventions. We analysed data encompassing antibiotics ordered by residents on hospital medicine teams in 2023 to identify the most common antibiotics requiring pharmacist intervention and for what reason, as displayed on Pareto charts (Figure 1) [13].

Using insights gained from the needs assessment and from the pharmacy repository, our multidisciplinary team of hospital medicine physicians, residents and pharmacists developed a website using the SquareSpace platform (www.cincyantibiotics.com). An institutional media designer was involved in website construction. The website was designed to align with just‐in‐time adult learning principles [14] by providing easily accessible, digestible information relevant to user needs. The tool was advertised to all paediatric residents and hospital medicine faculty and was designed as a standalone resource (not integrated into the EHR). Users could access the website independently, typically on mobile devices or workstations, as a just‐in‐time supplement to ordering decisions.

**FIGURE 2:**
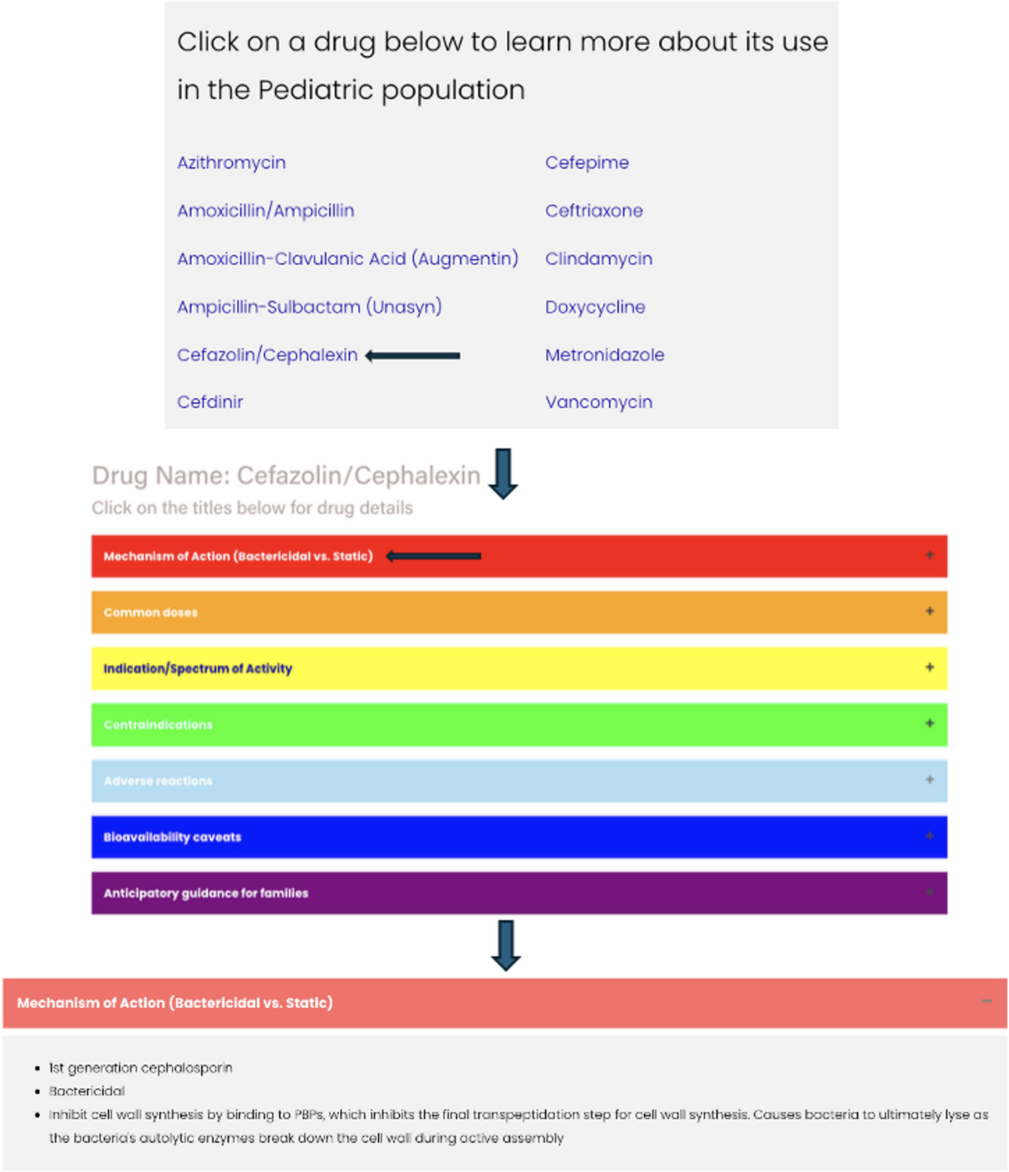
Screenshots illustrating the website homepage and an example antibiotic.

Based on pharmacy repository data, 13 antibiotics most frequently associated with pharmacist interventions were selected with content tailored to common prescribing errors (e.g., dosing regimens). Paediatric hospital medicine physicians, paediatric residents and pharmacists drafted all antibiotic content using existing institutional and published resources. Prior to publication, all content was reviewed by study team members and non‐team member inpatient infectious disease pharmacists for accuracy. Content updates are guided by primary study investigators J.B. and S.T.G. with new content reviewed by pharmacists prior to publication. Finally, the website and content review process were discussed with institutional legal representatives for authorisation for local dissemination. The website, launched in January 2024, features a homepage that featured a user‐friendly interface that invited users to select from a menu of 13 antibiotics. Each antibiotic page provided clear, concise information including mechanism of action, dosing guidance by indication, key contraindications, adverse reactions, bioavailability caveats (e.g., lung or central nervous system penetration) and anticipatory guidance for families. The website was formatted with collapsible sections optimised for both computer and mobile device browsing (Figure 2). Designed for rapid point‐of‐care consultation, users could rapidly find needed information and review clinical pearls before prescribing.

The website homepage directed users to identify themselves as paediatric residents versus guests (i.e., all others) given residents were the target population for our analysis. We monitored site traffic using SquareSpace analytics throughout the 6 months following website launch. We measured website usability using the Systems Usability Scale (SUS) [15], a validated scale for evaluating usability and acceptability of digital tools, 6 months after website launch via a voluntary, anonymous survey disseminated to paediatric residents. The SUS contains 10 survey items on a 5‐point Likert scale (*Strongly Disagree* to *Strongly Agree*), including alternating positive‐ and negative‐response items. Scaled scores range from 0 to 100 with data suggesting discrete categories for different ranges (e.g., 25–50 connotating a ‘poor’ tool, 70–80 being ‘good’ and > 80 being ‘excellent’ or ‘best imaginable’). Generally, scores above a 70 are ‘acceptable’ to users [15]. We gathered feedback through anonymous free‐response forms on the website and through free text responses in the post‐intervention survey.

## 3 Evaluation

The website was visited 1100 times over 6 months (average 46 visits per week), including 734 unique site visits by users, as defined by the host server, identifying themselves as paediatric residents (66.7%). The top 3 most common antibiotic pages visited by residents included cefazolin/cephalexin (139 visits), amoxicillin/ampicillin (113 visits) and amoxicillin‐clavulanate (112 visits).

Of 98 residents, 41 (42%) responded to the post‐survey, with 22 completing the SUS (Table 2) in its entirety. Participants rated the website as usable, with an average resident SUS score of 77.3 (SD = 14.3), indicating above‐average usability. In response to open‐ended questions soliciting feedback, residents reported the tool as ‘easier to use’ than the institutional medication database and reported the tool to be useful in guiding teaching. Formative feedback included requests for doxycycline as an additional antibiotic.

**TABLE 2:** System Usability Scale (SUS) items.

Positive response items (5 = *strongly agree*)	Median (IQR), *n* = 22
I think that I would like to use this website frequently.	4 (3–4)
I thought the website was easy to use.	4 (3.25–5)
I found the various functions in this website were well integrated.	4 (3–4)
I imagine that most people would learn to use this website very quickly.	4 (4–5)
I felt very confident using this website.	4 (4–4)

Negative response items (1 = *strongly disagree*)	Median (IQR)
I found the website unnecessarily complex.	2 (1–2)
I think that I would need the support of a technical person to be able to use this website.	1 (1–1.75)
I thought there was too much inconsistency in this website.	2 (1.25–2.75)
I found the website very awkward to use.	2 (1–2)
I needed to learn a lot of things before I could get going with this website.	1 (1–2)

*Note:* To calculate a final score: 1 point is subtracted from positive response items, and the negative response item scores are subtracted from 5. These scaled scores (0–4) are added up and multiplied by 2.5 to convert to a final SUS score between 0 and 100.

## 4 Implications

This study demonstrates the feasibility and early uptake by paediatric residents of a web‐based prescribing resource constructed from local prescribing data and resident input. High site traffic and above‐average usability ratings suggest that the tool was acceptable to users and could be integrated into existing clinical workflows.

While developed locally, the antibiotics included in the tool reflect paediatric prescribing patterns for common diagnoses, increasing its applicability to other training programs. Content can be tailored to local formularies or institutional guidelines, and the tool's design allows for adaptation across diverse clinical settings. Additionally, the development process (grounded in real‐world prescribing data, targeted needs assessment, and adult learning theory [8]) is a transferable model for other institutions aiming to create focused, learner‐centred medication educational tools, even beyond antibiotics. This approach offers a structured method for developing locally responsive prescribing resources grounded in institutional data. By leveraging real‐world prescribing data, incorporating resident input, and aligning content with adult learning principles [8], we ensured that our website directly addressed common prescribing errors and learning needs, enhancing the tool's relevance and impact.

High usability ratings indicate that residents found the tool easy to navigate and acceptable for clinical use. In contrast to textbooks or expansive databases, this institutional‐based tool allows for iterative modifications based on user feedback. This degree of adaptability underscores the importance of a ‘live’ resource that evolves alongside clinical needs. Other areas that worked well included the tool's clarity, accessibility and alignment with resident needs, as evidenced by strong engagement (site traffic) and positive feedback. Additionally, site traffic data demonstrated that resident engagement with specific antibiotic content aligned with prescribing challenges identified in the order intervention repository, as evidenced by the top two antibiotic groups identified by prescribing errors being the same top two antibiotic webpages visited most frequently by residents.

While this study did not directly evaluate for behavioural changes, this alignment suggests that real‐world prescribing difficulties may have led to resource utilisation. However, limitations included the lack of integration into clinical workflow (i.e., EHR), which may have reduced real‐time use during prescribing. Additionally, despite the SUS showing a high degree of reliability at variable sample sizes [15], the low survey response rate introduces potential bias, and the tool's impact on prescribing behaviours remains to be directly evaluated. However, nearly half of the residents completed aspects of the post‐survey, and both survey and unsolicited website feedback were positive. These factors, along with the high site traffic along the study period, suggest the tool's value extended beyond those who completed the SUS portion of the survey.

While our study used prescribing errors to guide content creation, future studies should prioritise embedding the website within the EHR to support point‐of‐care use and enable assessment of its impact on prescribing accuracy, antibiotic content knowledge and patient outcomes. This would allow for iterative improvements that address pertinent areas of need, along with linking the educational tool to patient outcomes. Broader implementation could benefit from strategies to support usage at the bedside and collaborate across institutions to expand content and reach and explore whether this intervention translates into measurable improvements in prescribing accuracy and patient safety.

In conclusion, our antibiotic tool addresses a practical gap in consolidating institutional‐specific antibiotic prescribing guidance into a concise, accessible format. Future work should evaluate the impact of such a resource on prescribing errors, pharmacist interventions and user knowledge.

*Our antibiotic tool addresses a practical gap in consolidating institutional‐specific antibiotic prescribing guidance into a concise, accessible format.*

## Author Contributions

**James Bowen:** conceptualization, investigation, writing – original draft, methodology, writing – review and editing, validation, formal analysis, data curation. **Johnathan Mollman:** investigation, writing – review and editing, methodology, validation, writing – original draft. **Daniel Herchline:** writing – original draft, methodology, writing – review and editing, validation. **Bindu Alex:** conceptualization, writing – review and editing, writing – original draft, methodology, validation. **Sarah Pickering:** conceptualization, writing – original draft, writing – review and editing, methodology, validation. **Sonya Tang Girdwood:** supervision, conceptualization, resources, investigation, writing – original draft, writing – review and editing, validation, methodology, formal analysis, data curation.

## Funding

This work was funded by internal institutional funding. Funders did not play a role in the conception or analysis of this study.

## Ethics Statement

This study was deemed exempt from full review by the local Institutional Review Board.

## Conflicts of Interest

The authors declare no conflicts of interest.

## Data Availability

The data that support the findings of this study are available on request from the corresponding author. The data are not publicly available due to privacy or ethical restrictions.

## References

[1] H. M. Salah , A. M. K. Minhas , M. S. Khan , et al., “Causes of Hospitalization in the USA Between 2005 and 2018,” European Heart Journal Open 1, no. 1 (2021): oeab001. 35919090 10.1093/ehjopen/oeab001 PMC9242058

[2] S. V. Kaiser , J. Rodean , E. R. Coon , S. Mahant , P. J. Gill , and J. K. Leyenaar , “Common Diagnoses and Costs in Pediatric Hospitalization in the US,” JAMA Pediatrics 176, no. 3 (2022): 316–318. 34962555 10.1001/jamapediatrics.2021.5171 PMC8715384

[3] Accreditation Council for Graduate Medical Education (ACGME) , “ACGME Program Requirements for Graduate Medical Education in Pediatrics [Internet],” Program Requirements, FAQs, and Applications, 2022, https://www.acgme.org/specialties/pediatrics/program‐requirements‐and‐faqs‐and‐applications/.

[4] “General Pediatrics Content Outline, In‐Training, Certification, and Maintenance of Certification Exams [Internet],” 2017, https://www.abp.org/sites/abp/files/gp_contentoutline_2017.pdf.

[5] M. M. Sattler , S. Greer , C. R. Lockowitz , J. G. Newland , E. E. Facer , and K. Wolfe , “Developing a Curriculum on Antimicrobial Stewardship for Pediatric Residents: A Needs Assessment,” Antimicrobial Stewardship and Healthcare Epidemiology 5, no. 1 (2025): e14. 39839360 10.1017/ash.2024.492 PMC11748016

[6] J. Martínez‐Domínguez , O. Sierra‐Martínez , A. Galindo‐Fraga , et al., “Antibiotic Prescription Errors: The Relationship With Clinical Competence in Junior Medical Residents,” BMC Medical Education 22, no. 1 (2022): 456. 35701813 10.1186/s12909-022-03499-0 PMC9199232

[7] J. D. Robinson and A. M. Persky , “Developing Self‐Directed Learners,” American Journal of Pharmaceutical Education 84, no. 3 (2020): 847512. 32313284 10.5688/ajpe847512 PMC7159015

[8] B. A. Mukhalalati and A. Taylor , “Adult Learning Theories in Context: A Quick Guide for Healthcare Professional Educators,” Journal of Medical Education and Curricular Development 6 (2019): 2382120519840332. 31008257 10.1177/2382120519840332 PMC6458658

[9] M. Schmitt , M. Hawkins , and P. Florsheim , “Key Determinants in Implementation Processes: A Systematic Review Using the Consolidated Framework for Implementation Research (CFIR),” Implementation Science Communications 6, no. 1 (2025): 89. 40846990 10.1186/s43058-025-00712-1 PMC12374266

[10] B. Rolland , F. Resnik , S. D. Hohl , L. J. Johnson , M. Saha‐Muldowney , and J. Mahoney , “Applying the Lessons of Implementation Science to Maximize Feasibility and Usability in Team Science Intervention Development,” Journal of Clinical and Translational Science 5, no. 1 (2021): e197. 34888066 10.1017/cts.2021.826 PMC8634288

[11] J. S. Holtrop , P. A. Estabrooks , B. Gaglio , et al., “Understanding and Applying the RE‐AIM Framework: Clarifications and Resources,” Journal of Clinical and Translational Science 5, no. 1 (2021): e126. 34367671 10.1017/cts.2021.789 PMC8327549

[12] D. Kern , P. Thomas , M. T. Hughes , and B. Chen , Curriculum Development for Medical Education: A Six‐Step Approach, 3rd ed., (Johns Hopkins University Press, 2016).

[13] A. Keniston , L. McBeth , G. Astik , et al., “Practical Applications of Rapid Qualitative Analysis for Operations, Quality Improvement, and Research in Dynamically Changing Hospital Environments,” Joint Commission Journal on Quality and Patient Safety 49, no. 2 (2023): 98–104. 36585315 10.1016/j.jcjq.2022.11.003

[14] E. S. Merriam , B. Sharan , R. S. Caffarella , and L. M. Baumgartner , “Learning in Adulthood: A Comprehensive Guide,” Journal of Adult Theological Education 5, no. 2 (2008): 202–203.

[15] A. Bangor , P. T. Kortum , and J. T. Miller , “An Empirical Evaluation of the System Usability Scale,” International Journal of Human Computer Interaction 24, no. 6 (2008): 574–594.

